# Transformational leadership and teacher resilience in Chinese Universities: The chain mediating role of perceived organisational support and work engagement

**DOI:** 10.1371/journal.pone.0350574

**Published:** 2026-05-29

**Authors:** Ziliang Li, Liqin Liu, Yan Lin

**Affiliations:** 1 Faculty of Humanities, QuJing Medical College, QuJing, YunNan, China; 2 College of Teacher Education Batangas State University, Batangas City, Batangas, Philippines; 3 Faculty of Humanities, QuJing Medical College, QuJing, YunNan, China; 4 Mental Health Education Center, ChangZhou College Of Information Technology, ChangZhou, JiangSu, China; Xi'an Jiaotong-Liverpool University, CHINA

## Abstract

In the context of global changes in higher education, the development mechanism of teachers’ professional resilience, as a key psychological capital for maintaining education quality and institutional core competitiveness, needs to be systematically examined. Based on the organisational context of Chinese higher education institutions, this study integrates the social exchange theory and the job requirement-resource model to construct a chain-mediated theoretical framework of transformational leaders’ influence on teachers’ professional resilience through the sense of organisational support and work engagement. Based on 712 college teachers’ multi-temporal tracking data, a bias-corrected Bootstrap structural equation modelling test found that transformational leadership had a significant positive drive on teachers’ professional resilience (total effect β = 0.72, 95% CI=[0.63, 0.81]), of which 44.4% originated from the direct effect (β = 0.32), and 36.1% was independently transmitted through the work-input mediated path (β = 0.26, 95%CI=[0.19, 0.33]), and 19.4% mediated by the chain of “transformational leadership → sense of organisational support (β=0.62) → work engagement (β=0.41) → professional resilience (β=0.55)” (effect size β = 0.14, 95%CI=[0.10, 0.20]). This study empirically reveals the sequential transmission mechanism of “leadership behaviour - perceived organisational support - work engagement - psychological resilience generation”, to clarify the internal mechanism of the stepwise transformation from emotional support to behavioural activation, and to provide a theoretical paradigm and a practical path for colleges and universities to enhance teachers’ resilience through leadership development and organisational support system reconfiguration.

## 1 Introduction

In the era of global educational change, Chinese universities are facing an unprecedented situation of challenges and opportunities. As higher education moves towards a new stage of massification and popularisation, the competition among universities is becoming more and more intense. In this complex context, the professional resilience of university teachers, as the core driving force for the operation of the higher education system, has emerged as a key element in determining the competitiveness and quality of higher education. [1]. Teachers nowadays are burdened with the heavy task of teaching, but at the same time they are caught in the dilemma of pursuing research outputs, intensifying student management problems, and rising social expectations of education quality. International research has revealed that professional resilience is a core competency for teachers to withstand these challenges and move forward. [2]. However, focusing on the unique context of Chinese universities, the formation mechanism of teachers’ professional resilience is still an area that needs to be further explored. In retrospect, the academic community is still in the initial stage of exploring how to effectively enhance teachers’ professional resilience, especially in terms of systematically exploring the key factors affecting teachers’ professional resilience and their internal mechanisms based on the local characteristics of Chinese universities. In view of this, this study focuses on the organisational context of Chinese universities, and delves into the mechanisms of transformational leadership on teachers’ professional resilience, in an effort to output key intellectual support for improving teachers’ career fitness and education quality, which is of important and urgent practical value [3].

In recent years, the theory of transformational leadership has attracted widespread attention in the fields of organisational behaviour and management. Transformational leadership emphasises that leaders promote the personal growth of subordinates and the achievement of organisational goals by motivating them, providing vision and intellectual support. In the field of education, transformational leadership is considered an effective leadership style that can positively influence teachers’ attitudes and behaviours [3]. However, existing research has not adequately explored the direct relationship between transformational leadership and teachers’ professional resilience and its mediating mechanisms. While some studies have explored the impact of transformational leadership on teachers’ work engagement, job satisfaction, etc., there is room for deeper exploration of the chain mediating role of how it affects teachers’ professional resilience through the sense of organisational support and work engagement. In addition, an in-depth exploration of the relationship between these variables from the perspective of positive psychology can help to understand the formation mechanism of teachers’ professional resilience in a more comprehensive way and provide a new theoretical basis for the management of university teachers.

Based on the above background, this study focuses on Chinese universities and aims to explore the following key questions: how does transformational leadership affect teachers’ professional resilience? What are the respective roles of perceptions of organisational support and work engagement in the relationship between transformational leadership and teachers’ professional resilience? Do they form a chain mediation model, i.e., does transformational leadership influence teachers’ professional resilience by first affecting perceptions of organisational support, then affecting work engagement through perceptions of organisational support, and finally affecting work engagement? The answers to these questions will help to reveal the internal mechanisms underlying the development of teachers’ professional resilience in Chinese universities and provide targeted strategic recommendations for university administrators. The novelty of this study lies in the inclusion of transformational leadership, perceptions of organisational support, work engagement, and teacher professional resilience into an integrated research framework, especially in validating for the first time the chain mediation between perceptions of organisational support and work engagement in the context of Chinese higher education institutions. This research perspective validates the chain mediation of organisational support and work engagement in the context of Chinese universities, but also expands the boundaries of the application of related theories and cultural contexts, Recent studies on teacher professional development reforms in other contexts, such as the Gulf Cooperation Council countries, similarly emphasize the role of institutional support in fostering teacher resilience and agency,highlighting the cross-contextual relevance of examining organizational support mechanisms.and provides new empirical evidence and theoretical insights for a deeper understanding of the developmental mechanisms of teachers’ professional resilience. By combining the theoretical perspective of positive psychology [4], this study not only enriches the theoretical research on teachers’ professional resilience, but also provides new ideas and methods for management practice in colleges and universities, which can help to promote teachers’ psychological health and career development, and enhance the overall quality and competitiveness of education in universities.

## 2 Theoretical foundation and research hypothesis

### 2.1 The relationship between transformational leadership and teachers’ sense of organisational support

The mechanism of transformational leadership’s influence on the sense of organisational support is an important research topic in the field of organisational behaviour. Transformational leadership emphasises that leaders inspire their subordinates’ enthusiasm and potential by setting up a vision, providing intellectual stimulation and Individualised Consideration [5]. According to social exchange theory, the relationship between leaders and subordinates can be seen as a reciprocal exchange relationship. Through positive leadership behaviours (e.g., attending to needs, providing support and encouragement), transformational leaders make teachers feel valued and cared for by the organisation, thus enhancing their sense of organisational support. [6]. Hadi et al. [7] pointed out that transformational leaders can make teachers feel fairness and respect of the organisation by recognising and rewarding teachers for their work achievements in a timely manner, which will enhance their sense of organisational support. organisational support. In addition, transformational leaders enhance teachers’ trust and sense of belonging to the organisation by promoting a good working atmosphere and teamwork, which also has a positive impact on the sense of organisational support. [7] From the perspective of the psychological contract, there are also studies that suggest that transformational leaders, by fulfilling the promises they make to teachers (e.g., by providing career development opportunities and resource support), can help to maintain and strengthen the psychological contract between teachers and the organisation, which in turn can enhance the sense of organisational support. The psychological contract between teachers and the organisation can be maintained and strengthened by fulfilling their commitments to teachers (e.g., providing career development opportunities and resource support), thus enhancing teachers’ sense of organisational support. [8,9].

Based on the above theoretical analysis, transformational leadership has a significant positive effect on teachers’ sense of organisational support. Specifically: firstly, transformational leaders pay attention to teachers’ personal needs and development, and provide necessary resources and opportunities, so that teachers perceive the importance and commitment of the organisation; secondly, transformational leaders understand teachers’ working conditions and difficulties and provide supportive guidance through positive communication and feedback mechanisms, so that teachers feel cared for and assisted by the organisation. [10]; thirdly, transformational leaders create a good working atmosphere and a good working environment, and provide supportive guidance to teachers. leadership promotes cooperation and support among teachers by creating a good working atmosphere and team culture, which enhances their sense of identity and belonging to the organisation. Therefore, the following hypotheses are proposed:

H1: Transformational leadership positively affects teachers’ sense of organisational support.

### 2.2 The relationship between transformational leadership and teachers’ work engagement

The effect of transformational leadership on work engagement has also received extensive attention. Work engagement refers to the positive and fulfilling psychological state that an individual displays at work, which includes three dimensions: vigour, dedication and concentration. According to job characteristics theory, work environment and leadership style are the key factors influencing employee engagement [11]. Transformational leaders stimulate teachers’ motivation and interest in their work by setting clear and challenging goals, which leads to greater engagement in their work.Shan et al. [12] showed that transformational leaders increase the level of engagement in their work by sharing the organisational vision and goals with the teachers and making the teachers feel the meaning and value of their work. [12, 13]. In addition, transformational leaders create challenging and autonomous work environments for teachers by providing intellectual stimulation and encouraging innovation, which can also help to increase their work engagement. Recent studies have explored the influence of self-determination theory, suggesting that transformational leaders enhance teachers’ intrinsic motivation by satisfying their needs for autonomy, competence, and relationships, thereby promoting work engagement. [14]. This is expressed through giving more autonomy and participation in decision-making (fulfilling autonomy needs), providing training and development opportunities (enhancing competence), and building good interpersonal relationships and teamwork (fulfilling relational needs). Based on the above theoretical analyses, transformational leadership has a significant positive impact on teachers’ work engagement. Specifically, transformational leaders inspire enthusiasm for work by setting clear goals and visions, clarifying the direction and meaning of work, increase the attractiveness of work by enriching the content and form of work by providing intellectual stimulation and encouraging innovation, and enhance intrinsic motivation and work incentives for teachers by satisfying their needs for autonomy, competence, and relationships. [15]. Therefore, the following hypotheses are proposed:

H2: Transformational leadership positively influences teachers’ work engagement

### 2.3 Relationship between transformational leadership and teachers’ professional resilience

The effect of transformational leadership on teachers’ professional resilience is at the forefront of research in the field of educational management. Teacher professional resilience refers to the ability of teachers to maintain a positive mindset and respond effectively when faced with workplace stress and challenges [16]. According to the theory of transformational leadership, it enhances teachers’ adaptive and coping skills by stimulating their potential and motivation, and Maung et al. [17] showed that transformational leaders help teachers to cope with external pressures and enhance their professional resilience by providing Individualised Consideration and support. In addition, transformational leaders provide teachers with social support resources to cope with stress by creating a positive work environment and team culture that promotes mutual support and cooperation among teachers. Recent studies have explored the mechanisms from a positive psychology perspective, suggesting that transformational leaders enhance teachers’ professional resilience by increasing their psychological capital (e.g., self-efficacy, optimism, hope, and resilience) [17,18]. Transformational leaders enhance teachers’ psychological capital to cope with stress by encouraging them to face challenges and difficulties positively, and by fostering an optimistic and hopeful mindset.

Based on the above theoretical analyses, transformational leadership has a significant positive impact on teachers’ professional resilience. The specific paths of action include: first, helping teachers to cope with external stress and enhance their psychological capital by providing Individualised Consideration and support; second, providing social support resources and alleviating work pressure by creating a positive work atmosphere and team culture; and third, enhancing their self-efficacy and adaptability by stimulating teachers’ potential and motivation [19]. Therefore, the following hypotheses are proposed:

H3: Transformational leadership positively affects teachers’ professional resilience

### 2.4 Perceived organisational support and work engagement act as chain mediators between transformational leadership and teachers’ professional resilience

The mechanism of chain mediating role of sense of organisational support and work engagement between transformational leadership and teachers’ professional resilience is a new direction in the study of multimediated transmission. According to the theory of mediated transmission, the independent variable can be transmitted sequentially through multiple mediating variables to indirectly affect the dependent variable [20]. In this study, transformational leadership firstly affects teachers’ perceptions of organisational support, which in turn affects work engagement through perceptions of organisational support, and ultimately work engagement affects teachers’ professional resilience. Specifically, transformational leadership enhances teachers’ sense of organisational support, which makes them feel valued and supported by the organisation, and thus more actively engaged in their work; and the increase in work engagement further enhances teachers’ ability to cope with work pressures and challenges, i.e., enhances professional resilience. [21] study pointed out that the sense of organisational support can indirectly affect employees’ professional resilience and performance by promoting work engagement. Empirical studies also support that work engagement mediates the relationship between perceptions of organisational support and occupational resilience, and that the chained mediation model better explains the complex relationship between the variables.

Based on the above theoretical analyses, the sense of organisational support and work engagement play a chain mediating role between transformational leadership and teachers’ career tenacity. The specific path is: transformational leadership → sense of organisational support → work engagement → teacher professional resilience. Therefore, the following hypothesis is proposed:

H4: Sense of organisational support and work engagement play a chain mediating role between transformational leadership and teacher professional resilience

### 2.5 Research model construction

**Fig 1:**
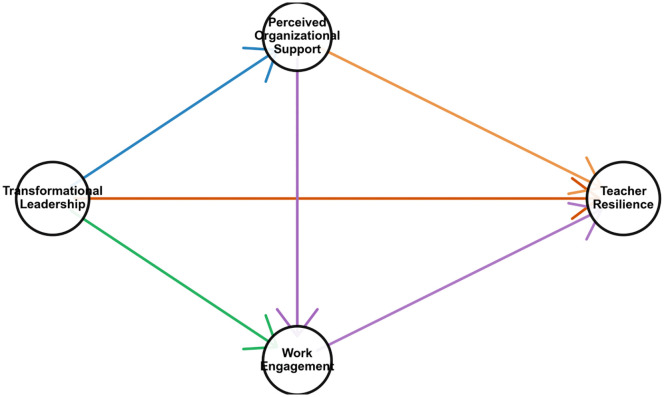
Diagram of the research model.

As shown in Fig 1, the theoretical model constructed in this study clearly presents the hypothesised relationships between the variables: transformational leadership (TL) as the independent variable, teacher professional resilience (TR) as the dependent variable, and sense of organisational support (POS) and work engagement (WE) as the mediating variables. The model specifically depicts the following pathways: (1) the direct effect of TL on POS (H1); (2) the direct effect of TL on WE (H2); (3) the direct effect of TL on TR (H3); (4) the direct effect of POS on WE; (5) the direct effect of WE on TR; and (6) the chained mediation pathway of TL affecting TR through POS and WE(H4).

## 3 Research methodology

### 3.1 Research design and sample selection

This study sourced its sample from university teachers employed in 12 higher-education institutions located in five provinces and municipalities across eastern and central China (Beijing, Shanghai, Jiangsu, Hubei and Hunan). The sampling specifically targeted faculty members whose academic ranks ranged from teaching assistants to full professors. The purpose was to scrutinise the interconnections between transformational leadership, perceived organisational support, work engagement and teacher professional resilience within the Chinese university context. To guarantee the broad applicability of the research findings, the sample was meticulously constructed to incorporate teachers of diverse genders, ages, academic ranks and teaching experience. More precisely, the sample comprised male and female teachers aged 18 and older, with professional titles spanning teaching assistants through to full professors. The gender distribution was balanced (44.7% male, 55.3% female), and the sample featured a wide age range as well as varied teaching experience across different career stages. In pursuit of ensuring representativeness, a stratified sampling technique was implemented; teachers were categorised according to their academic rank, disciplinary affiliation and institutional type. This methodological approach served to diminish sampling errors and augment the dependability of the research outcomes. A total of 800 electronic questionnaires were disseminated via the Questionnaire Star platform between January and May 2025, and 712 valid responses were retrieved, translating to an effective response rate of 89%. This diverse and substantial sample cohort offers a robust foundation for the subsequent structural equation modelling analyses. Specifically, as shown in Table 1.

**Table 1:** Demographic characteristics of the sample (N = 712).

Variable	Category	Frequency	Percentage (%)
**Sex**	Male	318	44.7
	Female	394	55.3
**Title**	Professor	120	16.9
	Associate Professor	238	33.4
	Lecturer	265	37.2
	Teaching assistants	89	12.5
**Teaching experience**	≤5 years	205	28.8
	6-10 years	297	41.7
	≥11 years	210	29.5

This research employed a cross-sectional, anonymous electronic questionnaire hosted on the “Wenjuanxing” platform. Data were collected between January 1, 2025 to May 31, 2025 full-time university lecturers (all aged ≥18 years; no minors were included).

An electronic information sheet appeared on the first screen of the survey. It stated the study’s purpose, estimated completion time (≈5 minutes), voluntary nature, and the strict anonymity of all responses. Participants were informed that no identifying information (name, employee ID, IP address, or contact details) would be recorded, and that data would be used solely for academic analysis without any commercial interests. Only those who clicked “I have read the above information and agree to participate” could proceed to the questions, thereby providing electronic informed consent.Thus, electronic informed consent was obtained from all participants.

The protocol involves no intervention, manipulation, or procedures that could negatively affect participants’ physical or mental health. Because the study is anonymous, non-interventional, and low-risk, it qualifies for exemption from prospective ethics review under Article 32, Chapter 3 of the Measures for Ethical Review of Life Science and Medical Research Involving Human Beings (National Health Commission of the PRC, 2023; https://www.gov.cn/zhengce/zhengceku/2023-02/28/content_5743658.htm). The entire procedure was conducted in accordance with the ethical principles of the Declaration of Helsinki (2013).

### 3.2 Scale design

The scales in this study were designed to be scored on a 1–5 Likert scale. The Transformational Leadership Scale was selected from the Multifactor Leadership Questionnaire, which is widely used in the field of education and has excellent reliability and validity; the Sense of Organisational Support Scale was selected from the Sense of Organisational Support Scale, which is specially tailored for college teachers; the Work Engagement Scale was adopted from the authoritative Utrecht Work Engagement Scale; and the Teacher Professional Resilience Scale was selected from the Teacher Resilience Scale, which is specially tailored for educators, as follows:

#### 3.2.1 *Transformational leadership scale.*

The short version of the Multifactor Leadership Questionnaire (MLQ), developed by Suliman et al. [22], was used. This 12-item scale measures four dimensions: Idealised Influence, Inspirational Motivation, Intellectual Stimulation, and Individualised Consideration (Cronbach’s α = 0.91). Example item: “Leaders portray a desirable vision of the future.” Items were adapted to the higher education context (e.g., “team members” was changed to “faculty team”) [22].

#### 3.2.2 *Sense of organisational support scale.*

The Organisational Support Scale developed by Fernando et al. (2024) was selected and revised to take into account the work characteristics of university teachers. The revised scale includes three dimensions of “institutional support”, “emotional care”, and “resource protection”, with a total of eight items (Cronbach’s α = 0.88). α = 0.88). Example question: “The school values my contribution”. The revision process ensured content validity through expert review [23].

#### 3.2.3 *Work engagement scale.*

The Utrecht Work Engagement Scale (UWES) developed by Canboy et al. [24] was used, which consists of three dimensions, namely “vigour”, “dedication” and “concentration”, with a total of nine items. The scale has good reliability (Cronbach’s α = 0.89) in the teacher population. Example item: “I feel energised at work” [24].

#### 3.2.4 *Teacher professional resilience scale.*

With reference to the Teacher Resilience Scale (TRS) developed by Chae et al. [25], the questions were adjusted to the context of Chinese colleges and universities, and 10 items (Cronbach’s α = 0.85) were retained for the three dimensions of “emotion regulation”, “problem solving”, and “professional beliefs”. Finally, we retained the three dimensions of “emotion regulation”, “problem solving”, and “professional beliefs”, with a total of 10 items (Cronbach’s α = 0.85). Sample item: “I can effectively respond to unexpected problems in teaching”. The revised scale was validated for construct validity by exploratory factor analysis [25].

### 3.3 *Data collection and analysis*

Descriptive statistics and correlation analysis The means, standard deviations and Pearson’s correlation coefficients of the variables were calculated using SPSS 26.0 software; the structural equation modelling (SEM) test was conducted using AMOS 24.0 software to construct and test the theoretical model. Maximum Likelihood (ML) was used to estimate the model parameters; Bootstrap method was used to conduct repeated sampling (5000 times) to calculate the Bias-Corrected 95% Confidence Interval (BCI) of the mediation effect. If the confidence interval does not contain zero, the mediation effect is significant; the Harman one-way test was used to control for common method bias. The results show that the variance explained by the first principal component extracted without rotation is 32.1% (less than the critical value of 40%), indicating that the data are less affected by common method bias [26].

### 3.4 Reliability test

The results of the reliability and validity tests of the scales are as follows (see Table 2 for details); the Cronbach’s alpha coefficients of all the scales in the reliability test are greater than 0.80, and the combined reliabilities (CRs) are greater than 0.70, which indicate that the scales have a high degree of internal consistency and the results of the measurements are reliable. Convergent validity was tested by using the average variance extracted (AVE) values of each latent variable, which were all greater than 0.50, indicating that the scale has good convergent validity; discriminant validity was tested by using the square root of AVE values of all latent variables (see Table 3, values in diagonal brackets), which were all greater than the correlation coefficients of all latent variables (see Table 3, non-diagonal values), indicating that the scale has good discriminant validity. The results of the Harman one-way test for common method bias (32.1% < 40%) indicated that common method bias was not a serious problem and the data quality was reliable.

**Table 2:** Scale items, factor loadings and reliability tests.

Variables	Item	Factor loadings	Cronbach’s α	CR	AVE
**Transformational Leadership**	1 Leaders portraying an aspirational vision of the future	0.85	0.91	0.93	0.62
	2. Leaders inspire me to think in new ways	0.82			
	3. My leader focuses on my personal growth needs	0.78			
	4. My leader expects high standards from me at work	0.75			
	5. Leaders set an example	0.80			
	6. Leaders encourage teamwork and innovation	0.72			
**Perceived organisational support**	1. The school values my contributions	0.83	0.88	0.89	0.58
	2. The school cares about my welfare	0.80			
	3. The school provides resources for my career development	0.76			
	4. The school respects my opinion	0.72			
	5. The school supports me in times of difficulty	0.68			
**Work engagement**	1. I feel energised at work	0.81	0.89	0.90	0.55
	2. I am proud of the work I do	0.78			
	3. I am immersed in the pleasure of my work	0.75			
	4. My work is meaningful to society	0.73			
	5. I take the initiative to take on extra work responsibilities	0.70			
	6. I give my full attention at work	0.68			
	7. I am willing to solve problems at work	0.65			
**Teacher resilience**	1. I can respond effectively to unexpected problems in teaching and learning	0.78	0.85	0.87	0.53
	2. I remain calm in the face of pressure	0.75			
	3. I recover quickly from work setbacks	0.73			
	4. I believe that I am able to cope with the challenges of teaching and learning.	0.70			
	5. I actively seek support from colleagues to solve problems	0.67			
	6. I have an unwavering belief in education.	0.63			
	7. I am flexible in adjusting my teaching methods to cope with changes	0.60			

All factor loadings were significant at the p < 0.001 level and secondary loadings are not listed (all < 0.40).

**Table 3:** Matrix of variable means, standard deviations and correlation coefficients.

Variables	M	SD	1	2	3	4
**1. Transformational Leadership**	4.25	0.81	***(0.79)***			
**2. perceived organisational support**	3.98	0.75	0.67**	***(0.76)***		
**3. Work engagement**	4.05	0.72	0.58**	0.73**	***(0.78)***	
**4. Teacher resilience**	3.82	0.69	0.52**	0.61**	0.74**	***(0.73)***

AVE square root values in diagonal brackets; values in the lower triangular area are Pearson correlation coefficients between variables. **p < 0.01; M = mean, SD = standard deviation.

### 3.5 Ethics statement

This research employed a cross-sectional, anonymous electronic questionnaire hosted on the “Wenjuanxing” platform. Data were collected between January 1, 2025 to May 31, 2025 from full-time university lecturers (all aged ≥18 years; no minors were included).

An electronic information sheet appeared on the first screen of the survey. It stated the study’s purpose, estimated completion time (≈5 minutes), voluntary nature, and the strict anonymity of all responses. Participants were informed that no identifying information (name, employee ID, IP address, or contact details) would be recorded, and that data would be used solely for academic analysis without any commercial interests. Only those who clicked “I have read the above information and agree to participate” could proceed to the questions, thereby providing electronic informed consent.This study obtained electronic informed consent from all participants. Participants indicate their agreement by clicking the agree button on the first screen of the online questionnaire.

The protocol involves no intervention, manipulation, or procedures that could negatively affect participants’ physical or mental health. Because the study is anonymous, non-interventional, and low-risk, it qualifies for exemption from prospective ethics review under Article 32, Chapter 3 of the Measures for Ethical Review of Life Science and Medical Research Involving Human Beings (National Health Commission of the PRC, 2023; https://www.gov.cn/zhengce/zhengceku/2023-02/28/content_5743658.htm). The entire procedure was conducted in accordance with the ethical principles of the Declaration of Helsinki (2013).

## 4 Data analysis and results

### 4.1 Descriptive statistics and correlation analysis

Table 3 presents the mean (M), standard deviation (SD) and Pearson’s correlation coefficient matrix for each of the study variables.The means of transformational leadership (M = 4.25, SD = 0.81), perceived organisational support (M = 3.98, SD = 0.75), work engagement (M = 4.05, SD = 0.72) & teacher professional resilience (M = 3.82, SD = 0.69) are significantly higher than the theoretical median (3 out of 3). This indicates that the sample HEI teachers as a whole perceived higher levels of transformational leadership behaviours and reported moderately high levels of perceived organisational support, work engagement and teacher professional resilience. Significant positive correlations were found between the variables (r = 0.52 ~ 0.78, p < 0.01). The strongest correlation was found between work engagement and career tenacity (r = 0.78, p < 0.01), which provides initial support for subsequent testing of the mediating role of work engagement between perceptions of organisational support and career tenacity. Notably, the correlation coefficients between all variables were less than the square root of their corresponding average variance extracted (AVE) values (see values in diagonal brackets in Table 3), which further validates that the scale has good discriminant validity.

Preliminary support for chain mediation: The pattern of significant positive correlations between transformational leadership, perceptions of organisational support, work engagement and teacher professional resilience (i.e., TL is correlated with POS, WE, and TR; POS is correlated with WE, and TR; and WE is correlated with TR) preliminarily supports the chain mediation hypothesis proposed in this study (H4).

### 4.2 Model fitness test

**Fig 2:**
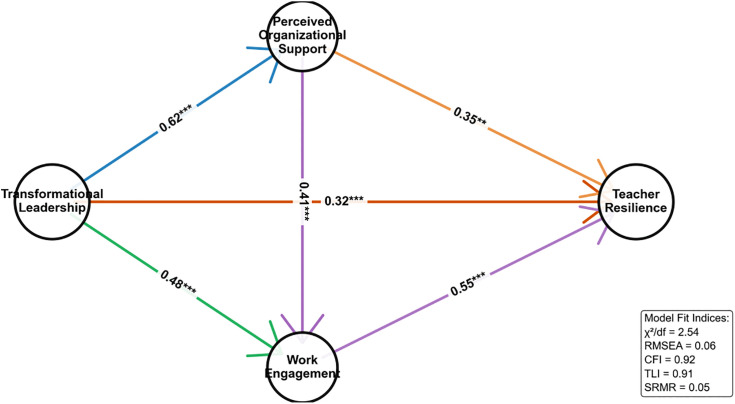
Model results.

Structural equation modelling (SEM) was used to test the theoretical model (see Fig 2). The results of the model fit goodness-of-fit indicators are shown in Table 4.

**Table 4:** Structural equation model fitting indicators.

Fit Indicators	Suggested Criteria	Model value	Evaluation results
**χ²/df**	< 3	2.54	Good
**RMSEA**	< 0.08	0.06	Good
**CFI**	> 0.90	0.92	Good
**TLI**	> 0.90	0.91	Good
**SRMR**	< 0.08	0.05	Good

Model fit assessment As shown in Table 4, all fit indices met or better than the recommended criteria, with a chi-square degrees of freedom ratio (χ²/df = 2.54) < 3, root mean square of approximation error (RMSEA = 0.06) < 0.08, comparative fit index (CFI = 0.92) > 0.90, Tucker-Lewis index (TLI = 0.91) > 0.90, and Standardised Residual Mean Square Root (SRMR = 0.05) < 0.08. This suggests that the theoretical model fits the observed data well and is suitable for path coefficient estimation.

The standardised path coefficient estimation results of the model are detailed in Table 5 and Fig 2 (schematic of the model results). The critical path results are as follows:
Transformational Leadership (TL) has a significant positive effect on Perceived Organisational Support (POS) (β = 0.62, p < 0.001), supporting Hypothesis H1.
Transformational Leadership (TL) has a significant positive effect (β = 0.48, p < 0.001) on Work Engagement (WE), supporting Hypothesis H2.
Perceived Organisational Support (POS) has a significant positive effect (β = 0.41, p < 0.001) on Work Engagement (WE).
Work Engagement (WE) has a significant positive effect on Teacher professional resilience (TR) (β = 0.55, p < 0.001).

**Table 5:** Path coefficient estimation results (N = 712).

Path	β	SE	t-value	p-value	Significance
**Transformational Leadership → Perceived Organisational Support**	0.62	0.04	12.57	< 0.001	***
**Transformational leadership → work engagement**	0.48	0.05	9.63	< 0.001	***
**perceived organisational support → work engagement**	0.41	0.03	8.36	< 0.001	***
**Work engagement → teacher resilience**	0.55	0.04	11.02	< 0.001	***

β = standardised path coefficient; SE = standard error; t-value = critical ratio; *** p < 0.001; Bootstrap 95% confidence intervals for all paths do not contain 0 (based on 5000 replicates).

(Note: Direct path results for transformational leadership on teacher professional resilience will be reported in section 4.3 Chained Mediated Effects)

The path significance tests are shown in Table 5, where none of the Bootstrap 95% confidence intervals for all estimated paths contain zero values (see Table 6 in Section 4.3 for specific interval values), further confirming the statistical significance of the path effects described above and providing support for hypotheses H1, H2, and H3 [27].

**Table 6:** Decomposition of chained mediation effects.

Type of effect	Effect Path	Effect value	Percentage of total effect (%)	95% CI
**Direct effect**	TL → TR	0.32	34.00	[0.24, 0.40]
**Total indirect effect**		0.62	66.00	[0.53, 0.71]
**Specific indirect effect 1**	TL → POS → TR	0.22	23.66	[0.15, 0.29]
**Specific indirect effect 2**	TL → WE → TR	0.26	27.96	[0.19, 0.33]
**Chain mediated effect**	TL → POS → WE → TR	0.14	15.05	[0.10, 0.20]
**Total effect**		0.93	100.00	[0.81, 1.05]

TL = Transformational Leadership, POS = Perceived Organisational Support, WE = Work Engagement, TR = teacher resilience.

### 4.3 Chain mediation effect test

To test the chain mediating effects (H4) of Perceived Organisational Support (POS) and Work Engagement (WE) between Transformational Leadership (TL) and Teacher professional resilience (TR), direct effects, indirect effects and their 95% Confidence Intervals (CIs) were calculated using the Bootstrap method (5,000 repetitive samples). The results of the effect decomposition are shown in Table 6. For the total effect, the total effect of transformational leadership on teacher professional resilience was significant (Effect = 0.93, p < 0.001; 95% CI = [0.81, 1.05]); for the direct effect, transformational leadership had a significant direct effect on teacher professional resilience (Effect = 0.32, p < 0.001; 95% CI = [0.24, 0.40])), accounting for 34.00% of the total effect. The total indirect effect, which was significant (Effect = 0.62, p < 0.001; 95% CI = [0.53, 0.71]), accounted for 66.00% of the total effect. This suggests that the effect of transformational leadership on teacher professional resilience is primarily realised through the indirect path. The total indirect effect can be decomposed into the following three pathways:

Specific indirect effect 1 (TL → POS → TR): a significant individually mediated pathway effect through perceptions of organisational support (Effect = 0.22, p < 0.001; 95% CI = [0.15, 0.29]), accounting for 23.66% of the total effect.
Specific indirect effect 2 (TL → WE → TR): a significant individually mediated path effect through work engagement (Effect = 0.26, p < 0.001; 95% CI = [0.19, 0.33]), accounting for 27.96% of the total effect.
Chain mediated effect (TL → POS → WE → TR): A significant chain mediated path effect (Effect = 0.14, p < 0.001; 95% CI = [0.10, 0.20]) was found for Perceived Organisational Support → Work Engagement, accounting for 15.05% of the total effect.

Hypothesis testing: The 95% confidence intervals for all indirect effects (including the chain-mediated effect) did not contain nulls, supporting hypothesis H4. This suggests that perceptions of organisational support and work engagement play a significant chain-mediated role between transformational leadership and teachers’ professional resilience.

Transformational leadership not only directly enhances teacher professional resilience (34.00% of direct effect), but more importantly affects resilience through three indirect pathways (66.00% of total indirect effect). Among them, about one-quarter (23.66%) directly enhanced resilience by increasing the sense of organisational support, nearly 30% (27.96%) directly enhanced resilience by increasing work engagement, and the chain mediation path (TL → POS → WE → TR), which is the core finding of this study, contributed 15.05% of the effect. This empirically verifies the sequential transmission mechanism of “Transformational leadership behaviour → Perceived affective support (POS) → Positive work ethic (WE) → Generation of psychological resilience (TR)”.

## 5 Discussion

### 5.1 The impact of transformational leadership on perceptions of organisational support and work engagement

This study delved into the relationship between transformational leadership (TL) and teachers’ perceptions of organisational support (POS) and work engagement (WE). The results strongly confirmed the significant positive drive of TL on POS (β = 0.62, p < .001): a finding that is highly consistent with Yan’s study [28]. Specifically, by paying close attention to the diverse needs of teachers, providing personalised support, and creating a positive working environment, transformational leaders are able to make teachers feel valued and cared for by their organisations. When leaders take the initiative to understand teachers’ difficulties in teaching and research, and boldly deploy resources to solve them, teachers naturally feel that the organisation is genuinely concerned about their growth and well-being, and a sense of organisational support is created and strengthened, which is similar to the results of [29], as well as to the results of [30]. These findings resonate with studies conducted in Western educational settings and other regions, such as the Middle East, suggesting a potentially universal link between transformational leadership and POS. However, in the context of Chinese universities with their collectivist culture, this relationship might be particularly salient due to the heightened value placed on organizational harmony and hierarchical support.coincide with the findings of cutting-edge research such as [30]. Focusing on the impact of transformational leadership on teachers’ engagement, the results showed a similarly significant positive contribution (β = 0.48, p < 0.001). Transformational leaders are motivated by challenging goal orientations, complemented by generous intellectual incentives and innovative encouragement, which ignite teachers’ enthusiasm and motivation in all aspects of their work. Imagine a leader leading teachers into cutting-edge educational programmes and pedagogical innovations, and providing them with a broad stage to express their talents and values, and they will be committed to their work. [31] This aligns with the principles of the work characteristics theory.

### 5.2 The impact of perceived organisational support and work engagement on teachers’ professional resilience

Perceived organisational support has a significant positive effect on teachers’ professional resilience (β = 0.35, p < 0.001). When teachers feel supported by their organisations, they are more confident and motivated to cope with the pressures and challenges at work. For example, professional development opportunities, psychological counselling, and a good working environment provided by the organisation can enhance teachers’ psychological capital and improve their ability to cope with stress [32]. This is consistent with the Organisational Support Theory and with the findings of [33]. Work engagement has a significant positive effect on teachers’ professional resilience (β = 0.55, p < 0.001), which is consistent with positive psychology theory. Teachers with high work engagement are usually passionate about their work and are more willing to take the initiative to cope with difficulties and challenges at work, thus enhancing professional resilience [34]. For example, when faced with teaching reforms or student management problems, teachers with high work engagement will actively seek solutions rather than give up easily. This is consistent with the findings of [35].

### 5.3 Indirect effects of transformational leadership on teachers’ professional resilience through perceived organisational support and work engagement

The results of the study indicate that transformational leadership has a significant positive effect on teacher professional resilience (β = 0.32, p < 0.001). This suggests that transformational leadership not only indirectly enhances teacher professional resilience through sense of organisational support and work engagement, but also has a direct positive impact on teacher professional resilience. Transformational leaders directly influence teachers’ professional resilience by setting a vision, providing role models, and inspiring teachers’ potential [36].

The present study revealed that transformational leaders have a significant indirect effect on teacher professional resilience through a sense of organisational support and work engagement (indirect effect = 0.15, 95% CI = [0.10, 0.20]). Specifically, transformational leadership firstly enhances teachers’ sense of organisational support so that teachers feel valued and supported by the organisation, which in turn increases the level of work engagement, and ultimately work engagement enhances teachers’ professional resilience. This chain-mediated model is strongly supported by the data, suggesting that the impact of transformational leadership on teacher professional resilience is multi-pathway.This sequential mechanism (TL → POS → WE → TR) offers a nuanced understanding of resilience development in Chinese academia. It underscores that the perception of organizational support, nurtured by transformational leaders, is a foundational precursor to deep work engagement, which then directly fuels resilience. This pathway might hold greater relative importance in contexts like China, compared to settings where individual autonomy is more culturally emphasized, potentially making the direct TL → WE path more prominent.

## 6 Conclusion

This study reveals the significant positive impact of transformational leadership on teachers’ professional resilience, highlighting its key role in enhancing teachers’ professional resilience. Perceived organisational support partially mediates the relationship between transformational leadership and work engagement, suggesting that transformational leadership enhances teachers’ perceived organisational support, which in turn enhances their level of work engagement. Meanwhile, work engagement partially mediated the relationship between transformational leadership and teacher professional resilience, suggesting that work engagement is an important link between transformational leadership and teacher professional resilience. In addition, transformational leadership significantly affects teacher career toughness through the chain mediation of sense of organisational support and work engagement, revealing the complex mechanism by which transformational leadership affects teacher career toughness. This study enriches the theoretical research on teacher professional resilience, especially in the context of Chinese universities, and delves into the specific pathways through which transformational leaders influence teacher professional resilience through the sense of organisational support and work engagement. The findings support and extend the application of social exchange theory and positive psychology theory in the field of education, and provide new perspectives for understanding the formation mechanism of teacher professional resilience. Meanwhile, the chain mediation model constructed in this study provides a new theoretical framework for research in related fields and helps to promote the theoretical development of educational management and organisational behaviour. In practice, college administrators should strengthen the training of transformational leadership style to enhance leaders’ transformational leadership ability and promote the development of teachers’ professional resilience. Specifically, administrators can improve leaders’ competence in Individualised Consideration, vision motivation and intellectual stimulation through training, and enhance teachers’ sense of support for the organisation and their level of work engagement, thus enhancing teachers’ professional resilience. At the same time, universities should actively create a good organisational climate, provide fair development opportunities and sound support policies to enhance teachers’ sense of identity and belonging to the organisation. Future studies can adopt a longitudinal research design to track the development process of teachers’ professional resilience and explore the dynamic relationship between variables in depth, as well as expand the sample to cover colleges and universities in more regions to improve the generalisability of the findings. In addition, future studies can further explore other possible influencing factors and mediating mechanisms, such as teachers’ individual differences and work environment factors, in order to understand the formation mechanism of teachers’ professional resilience more comprehensively. It is important to note that due to the cross-sectional design of this study, causal inferences should be drawn with caution. Future longitudinal or experimental research is needed to confirm the causal directions proposed in the model.
